# A complementary method for detecting qi vacuity

**DOI:** 10.1186/1472-6882-9-12

**Published:** 2009-05-08

**Authors:** Ming-Feng Chen, Hsi-Ming Yu, Shu-Fang Li, Ta-Jung You

**Affiliations:** 1 Department of Internal Medicine, Show Chwan Memorial Hospital, No.542, Sec 1, Chung-Shang Rd, Changhua City, Taiwan; 2 Department of Chinese Medicine, Tzu-Ai General Hospital, Hsilo, Yulin County, Taiwan; 3 Department of Clinical Laboratory Science, Tainan Municipal Hospital, Tainan City, Taiwan; 4 Institute of Chinese Medicine Science, China Medical University, Taichung City, Taiwan

## Abstract

**Background:**

Qi vacuity (QV) is defined by traditional Chinese medicine as a loss of energy in the human body. An objective method for detecting QV was not available until recently, however. The automatic reflective diagnosis system (ARDK) is a device that detects human bioenergy through measuring skin conductance at 24 special acupoints on the wrists and ankles.

**Methods:**

This study used the ARDK to measure skin conductance on 193 patients with QV and 89 sex- and age-matched healthy controls to investigate whether the device is useful in detecting QV. Patients diagnosed with QV have three or more of five symptoms or signs; symptom severity is measured on 5 levels and scored from 0 to 4 points. We compared the difference in the mean ARDK values between patients with QV and healthy controls, and further used linear regression analysis to investigate the correlation between the mean ARDK values and QV scores in patients diagnosed with QV.

**Results:**

The mean ARDK values in patients with QV (30.2 ± 16.8 μA) are significantly lower than those of healthy controls (37.7 ± 10.8 μA; *P *< 0.001). A negative correlation was found between the mean ARDK values and QV scores (*r *coefficient = -0.61; *P *< 0.001). After adjusting for age, the decreased mean ARDK values in patients with QV showed a significant correlation with the QV scores.

**Conclusion:**

These results suggest that the mean ARDK values reflect the severity of QV in patients diagnosed with the disorder. They also suggest that the bioenergy level of the human body can be measured by skin conductance. ARDK is a safe and effective complementary method for detecting and diagnosing QV.

## Background

Qi vacuity (QV) is a syndrome defined by traditional Chinese medicine as a loss of energy in the human body. Patients with QV commonly have symptoms of tiredness, general malaise, and loss of the ability to speak loudly or for long periods of time. Some patients with QV break abruptly into spontaneous sweating during movement. Practitioners of traditional Chinese medicine usually make the diagnosis of QV on the basis of subjective observation of the patient's symptoms and signs. A gold standard for diagnosing QV is not yet available, however. A professional committee formed by the Society of Integrated Chinese and Western Medicine for the study of qi vacuity patterns and age-related disease proposed a set of criteria for diagnosing QV [1]. The committee suggested that patients should have three or more of five major symptoms or signs to meet the diagnosis of QV. The five major symptoms and signs are: emotional fatigue with lack of strength; shortness of breath accompanied by reluctance to speak; spontaneous sweating; enlarged tongue with or without dental impressions; and a vacuous and weak pulse. The use of these criteria to make the diagnosis of QV is still subjective, however. The automatic reflex detecting system (ARDK) is an electrical device (Good News Natural Medicine Biotech Co. Ltd., Taichung, Taiwan) designed to detect skin conductance between the left hand and each of 24 special acupoints along the meridians on the wrists and ankles on both sides of the body. The mean values of skin conductance from these 24 acupuncture points are calculated automatically by a computer system; they reflect the energy levels in the body. It is not known whether ARDK can be used as an objective tool to diagnose QV. The aim of this study is to investigate whether the mean values of skin conductance as detected by ARDK will reflect the severity of QV in patients diagnosed with the disorder. We used ARDK equipment to measure skin conductance between the left hand and the 24 special acupoints in 193 patients previously diagnosed with QV and in 89 sex- and age-matched healthy controls. We compared the differences in the mean values of skin conductance between patients diagnosed with QV and healthy controls, and further analyzed the correlations between the mean values of skin conductance and the severity of QV in those patients.

## Methods

### Subjects

Eighty-nine healthy controls (33.7 +/- 10.3 years old, mean +/- SD) and 193 patients (30.2 +/- 16.8 years old) with a previous diagnosis of QV were enrolled in this study between August 1 and December 31, 2006.

The healthy controls (40 males and 49 females, ranging in age from 22 to 72 years) were selected from subjects who received a general health examination in the Health Examination Center of Tainan Municipal Hospital. They had no specific complaints during the previous month and no past history of chronic diseases, including diabetes; cardiovascular, respiratory, liver, renal, or autoimmune diseases; or malignancies. All had normal blood and urine values as well as normal EKG and serum biochemistry findings, including liver and renal function, blood sugar levels, and electrolytes.

The patients diagnosed with QV (83 males and 110 females, ranging in age between 20 and 72 years) were selected from outpatients in the Department of Integrated Chinese and Western Medicine in Tainan Municipal Hospital. All had major complaints of tiredness and general malaise. The QV subjects included 108 carriers of hepatitis B or C with normal liver function. There were no significant differences in sex and age between the QV patients and healthy controls.

### Study procedure

The study protocol was approved by the institutional review board of Tainan Municipal Hospital. All patients gave written informed consent. Each subject was then evaluated for the presence and severity of QV by two experienced practitioners of traditional Chinese medicine. A trained and experienced technician measured the subjects' skin conductance using an ARDK instrument in a room with a constant temperature of 25°C and humidity at 55%. The mean values of skin conductance in all subjects and the levels of severity in the QV patients were recorded and given to a statistician for analysis.

### Diagnosis and evaluation of QV

The QV syndrome was diagnosed according to the criteria proposed by the Society of Integrated Chinese and Western Medicine in China. Patients with three or more of the following symptoms or signs, including emotional fatigue with lack of strength; shortness of breath accompanied by reluctance to speak; spontaneous sweating; enlarged tongue with or without dental impressions; and a vacuous and weak pulse were diagnosed with QV. In this study, we further graded the severity of each symptom or sign on five levels scored from 0 to 4. The sum of the scores on the five symptoms or signs of QV denoted the severity of the disorder and was identified as the QV score.

### ARDK testing

The ARDK test was performed according to the standard procedure recommended by the manufacturer. Subjects were instructed to avoid the use of such caffeinated beverages as coffee or tea, or such drugs as beta-blockers, which may influence the activity of the sympathetic nervous system, for a period of 3 full days before the examination. Each subject was asked to rest and relax for a minimum of 20 minutes and to remove all jewelry and other metal accessories before the examination.

**Figure 1:**
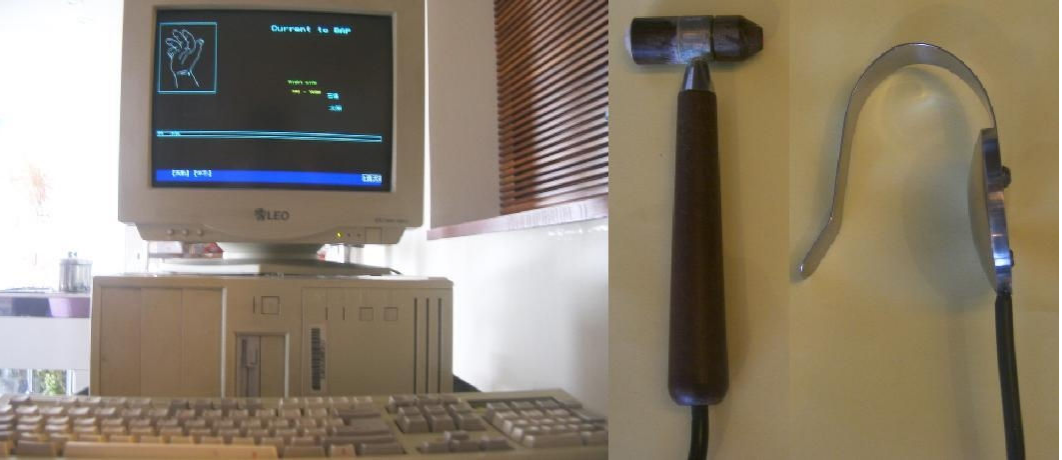
**ARDK equipment**. The ARDK equipment includes a metal plate, a detector, and the ARDK device. The metal plate and the detector are connected to the ARDK device, which supplies a weak electrical current with a constant voltage of 1.75V and is connected to a personal computer.

The subjects were then asked to lie on a bed and hold a metal plate in the left hand. Next, the technician used a small detector to measure the skin conductance over each of the 24 special acupoints in both wrists and ankles of each subject in sequence. Most of the chosen acupoints are the originating acupoints of the 12 meridians: LU9, PC7, HT7, SI4, SJ4, LI5, SP3, LR3, KI3, BL65, GB40, and ST40 in the wrists and ankles bilaterally. The ARDK equipment is shown in Figure 1; it includes a handheld metal plate, a detector, and the ARDK device. The metal plate is used to connect the left hand of the subject and the detector touching the subject's acupoints. The metal plate and detector are both connected to the ARDK device, which supplies a weak electrical current with a constant voltage of 1.75V and records about 50 values within 2 seconds of touching the skin. This device was also connected to a personal computer that automatically calculated the mean values of skin conductance of the 24 acupoints after the examination.

### Statistical analysis

The difference between the skin conductance of healthy controls and that of patients with QV was analyzed by Student's *t*-test with adjustment for alpha inflation. Linear regression analysis was used to investigate the correlation between skin conductance and age in the normal controls, and the correlation between the decrease in skin conductance and the QV score in the diagnosed patients. Further multiple regression analysis of the decrease in skin conductance using the scores of QV symptoms or signs as independent variables was performed by the least squares method.

## Results

### Mean total QV scores and mean value of ARDK in patients with QV and in healthy controls

The mean total QV score in patients diagnosed with the disorder (7.9 ± 5.3 points) is significantly greater than the scores of healthy controls (1.5 ± 1.4 points; *P *< 0.001). In contrast, the mean ARDK value in patients with QV (30.2 ± 16.8 μA) is significantly lower than that of healthy controls (37.7 ± 10.8 μA; *P *< 0.001). On the other hand, there is no significant difference in the mean ARDK value between males (38.2 ± 10.2 μA) and females (36.6 ± 10.5 μA) in healthy controls. Similarly, there is no significant difference in the mean ARDK value between males (31.2 ± 15.2 μA) and females (30.4 ± 13.5 μA) in patients diagnosed with QV.

### Correlation between mean ARDK value and age in healthy controls

**Figure 2:**
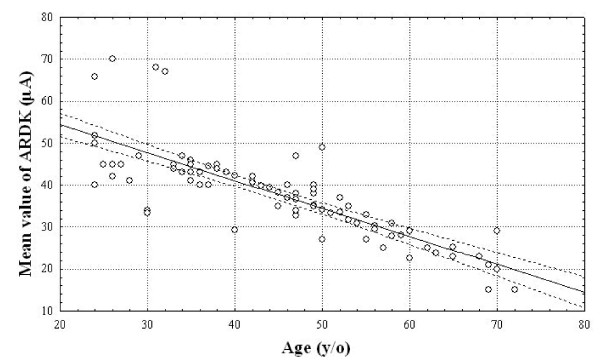
**Correlation between mean ARDK values and age in 89 healthy controls**.

As shown in Figure 2, the mean ARDK value in healthy controls decreases gradually with age. There is an inverse correlation between the mean ARDK value and age (*r *coefficient = -0.7; *P *< 0.001) with the following equation: mean ARDK value = 69 - (0.8 × age).

### Correlation between mean skin conductance values and QV scores

**Figure 3:**
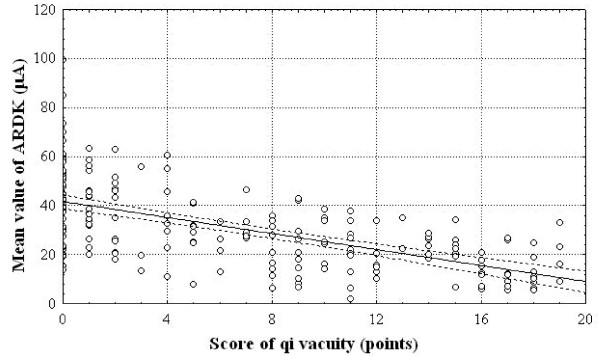
**Correlation between mean ARDK values and QV scores in 193 patients with QV**.

Figure 3 shows that the mean ARDK value decreases with an increase in the QV score of the observed patients. There is an inverse correlation between the mean ARDK values and the QV scores (*r *coefficient = -0.61; *P *< 0.001).

### Correlation between decreased ARDK value and QV scores

**Figure 4:**
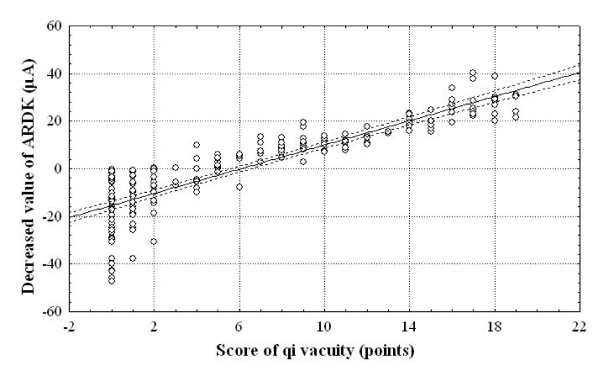
**Correlation between decrease in ARDK values and QV scores in 193 patients with QV**.

The decrease in ARDK value is calculated by subtracting the real mean ARDK value in each studied patient from the expected ARDK value adjusted by age according to the equation described in Figure 2. There is a positive correlation between the decrease in ARDK value and the QV scores in the studied patients (*r *coefficient = 0.89; *P *< 0.001), as shown in Figure 4.

### Multiple regression analysis

Multiple regression analysis of the decrease in ARDK values with the scores of the five symptoms or signs of QV and age as independent variables is given in Table 1. Only the scores associated with a weak pulse (β coefficient = 0.422, P < 0.001) and fatigue (β coefficient = 0.317, *P *< 0.001) showed a significant correlation with a decrease in the ARDK value, whereas spontaneous sweating, weak voice, enlarged tongue with dental impressions, and age did not show significant correlations.

**Table 1:** Multiple regression analysis of the decrease in ARDK values in. patients with QV.

**Independent variables**	**β-coefficient**	**P value**
Weak pulse	0.422	< 0.001
Fatigue	0.317	< 0.004
Spontaneous Sweating	0.100	0.063
Weak voice	0.079	0.330
Enlarged tongue with dental impressions	0.045	0.360
Age	0.001	0.970

## Discussion

The skin electrical conductance test has been used widely by psychologists as an indicator of psychological stress [2-5]. Several studies have indicated that skin electrical conductance may be influenced by such multiple psychological factors as anxiety [6,7], phobia [8,9], and schizophrenia [10]. The skin electrical conductance test has also been used as a tool for biofeedback training [11].

The skin electrical conductance test (ARDK) used in this study is different from the traditional conductance test used by psychologists. Psychologists usually perform the test using two different fingers on one of the subject's hands. In contrast, ARDK is used to detect the skin electrical conductance between the left hand and each of 24 special acupoints in both wrists and ankles. Most of the special acupoints are the original points that express the energy of the 12 major meridians. The ARDK test is a modification of the electrodermal screening test (EDST) proposed by Voll, who used the level and the balance of skin conductance among various acupoints on the four limbs to evaluate the energy status of the human body [12,13]. EDST has been widely used by practitioners of traditional Chinese medicine in the evaluation of patients' energy conditions. The reliability of EDST in clinical application is still unknown, however. Most versions of EDST used in traditional Chinese medicine are inconvenient due to the narrowness of the detector and inability to calculate the mean values of skin conductance automatically.

We previously used a bioenergy detector (VGH-82A, Kolon, Taipei) to measure the skin conductance between both palms and soles of patients with QV in order to develop an objective method for diagnosing QV [14]. We found that the mean values of skin conductance between the palms of the hands and soles of the feet can reflect the severity of QV. On the other hand, skin conductance between the palms and soles is easily influenced by psychological stress. Moreover, the bioenergy detector that we used (VGH-82A) cannot calculate the mean value of skin conductance automatically. Due to the narrow width and sharp profile of the detector, it is not very user-friendly for either physicians or technicians. In contrast, the ARDK has a wide detector and a function that automatically calculates the mean value of skin conductance, making it easy and comfortable to use.

The present study shows that the mean total QV score in patients diagnosed with the disorder is significantly higher than that of healthy controls. In contrast, the mean ARDK values in patients with QV are significantly lower than those of healthy controls. These results indicate that patients with QV have higher QV scores but lower levels of skin conductance than healthy controls. The mean ARDK values in healthy controls decrease with increasing age, suggesting that natural aging is an important factor in the decrease of skin conductance in the human body over time. Although gender may be a factor that influences skin conductance, we did not find a significant difference in the mean ARDK values between males and females in either the healthy controls or the observed patients.

Our finding that the mean ARDK values in patients with QV have a significantly inverse correlation with QV scores suggests that patients with more severe QV have lower levels of skin conductance. The additional finding that the decreased mean value of ARDK after age adjustment in patients with QV is positively correlated with QV scores further indicates that the decrease in the mean ARDK values after age adjustment may reflect the severity of QV.

The reason underlying the decrease in the mean value of skin conductance with age in healthy humans and the further decrease in patients with QV is still unknown. Electrodermal activity (EDA) can be influenced by hydration of the epidermis [15], based on the activities of the sweat glands and their innervation by the sympathetic nervous system [16,17]. Sympathetic nerve activity (SNA) in the skin is primarily under the control of the central nervous system, and appears to be directed to both the sweat glands and to vascular smooth muscles, with the relative targeting being temperature-dependent [18-20]. It is therefore possible that the decrease in the mean ARDK values with age in healthy humans may be due to the degeneration of the sweat glands or a decrease in SNA during the aging process. Furthermore, the decreased skin conductance in patients with QV may be due to a decrease in sweat gland activity resulting from the attenuation of SNA.

The results of multiple regression analysis of the decreased mean value of ARDK with the scores of the five symptoms and signs of QV and age as independent variables show that only the scores for fatigue and weak pulse contribute to the decrease in the mean ARDK values. Fatigue is a common symptom in patients with chronic diseases, including cystic fibrosis and Behçet's disease. A decrease in skin conductance has been reported in patients with cystic fibrosis [21] and Behçet's disease [22]. These results are similar to our findings that skin conductance is lower in patients complaining of tiredness, and is inversely correlated with the severity of QV. The significant correlations between skin conductance and fatigue or weak pulse may be due to a decrease in sympathetic nervous activity. The latter can not only induce a decrease in skin conductance via reduced secretions of the sweat glands but can also induce fatigue and weak pulse resulting from a reduction in the contractility of the left ventricle of the heart, according to the Frank-Starling law of the heart.

It is not yet known why the scores for such other symptoms of QV as spontaneous sweating, weak voice, and enlarged tongue do not contribute to the decrease in skin conductance in patients with QV. In general, patients with QV may have spontaneous sweating, which can increase skin conductance. In this study, however, we did not find a significant correlation between the scores for spontaneous sweating and the decrease in the mean ARDK values. The discrepancy may be due to the low incidence of spontaneous sweating in the subjects and their condition during ARDK measurement. Abrupt spontaneous sweating usually appears during movement in patients with severe QV. Only 23% of our subjects complained of spontaneous sweating during movement. None of them had an occurrence of spontaneous sweating during ARDK measurement, which was performed with the subjects in a relaxed condition lying on a bed after resting for a minimum of 20 minutes. The fact that skin conductance in patients with QV did not correlate with the scores for weak voice or enlarged tongue may be due to a more complex mechanism that induces these two symptoms rather than a simple decrease in sympathetic nervous activity.

It is possible that a change in skin conductance along a specific meridian could represent a specific illness. In this study, most of the patients diagnosed with QV were carriers of hepatitis B or C with normal liver function. We compared the skin conductance of the liver meridian with those of the other11 meridians but found no significant difference (data not shown). Therefore, we could not prove the above hypothesis.

There have been no reports in the literature to date describing a device that can be used as a tool to help in detecting and diagnosing QV. Our results suggest that the bioenergy of the human body can be measured by skin conductance. It also suggests that ARDK may be used as a complementary method for physicians to make a diagnosis of QV in clinical practice.

## Conclusion

A novel method, namely ARDK, was used to detect skin conductance between the left hand and each of 24 special acupoints in patients with QV and healthy controls. The QV patients were further evaluated for the severity of five major signs or symptoms with a five-point scoring system. Our results show that the mean value of ARDK decreases with increasing age in healthy controls and further decreases in patients with QV. The decrease in the age-adjusted mean ARDK values in patients with QV has a significant positive correlation with the severity of the disorder. Our results suggest that ARDK can be used as a complementary method to detect the presence and severity of QV.

## Competing interests

The authors declare that they have no competing interests.

## Authors' contributions

MC designed the experiment, recruited subjects, and performed the statistical analysis. HY and TY made the diagnoses of QV and evaluated the severity of the disorder in patients according to the criteria of traditional Chinese medicine. SL performed the ARDK examination of the subjects.

## Pre-publication history

The pre-publication history for this paper can be accessed here:


